# The Critical Moderating Role of Cognitive Function in Digital Inclusion: Data Analysis Study on Depression Risk Among Older Adults

**DOI:** 10.2196/80700

**Published:** 2025-11-25

**Authors:** Gang Xiao, Tingting Nie

**Affiliations:** 1 School of Mathematics and Statistics, Hanshan Normal University, Chaozhou, Guangdong province, China; 2 Department of Radiology, Hubei Cancer Hospital, Tongji Medical College, Huazhong University of Science and Technology, No 116 Zhuodaoquan South Load, Hongshan District, Wuhan, Hubei, 430079, China, 86 027-87670277

**Keywords:** cognitive aging, cognitive function, depression, digital inclusion, moderation effect, multiple pathways, older adults

## Abstract

**Background:**

Digital inclusion has become increasingly important in promoting healthy aging, yet its association with mental health among older adults appears complex and heterogeneous. The role of cognitive function as a moderator and the underlying mechanisms remain understudied.

**Objective:**

This study aims to examine cognitive function’s moderating role in the relationship between digital inclusion and depression risk among older adults, and to investigate multiple pathways of association.

**Methods:**

Using data from the 2020 wave of the China Health and Retirement Longitudinal Study, we analyzed 18,673 adults aged 60 years and above (mean age 68.4 y, SD 6.5; 50.8% male participants [n=9486], 49.2% female participants [n=9187]). We constructed interaction effect models to test the moderation hypothesis and employed path analysis with bootstrapped 95% confidence intervals (2000 iterations) to investigate multiple pathways through which digital inclusion is associated with depression.

**Results:**

Cognitive function significantly moderated the digital inclusion-depression relationship (*β*=−.002, *P*=.03). The association was not statistically significant at low cognitive function (*β*=−.137, *P*=.33) but strongly protective at high cognitive function (*β*=−.517, *P*<.001), revealing a “cognitive threshold effect.” Path analysis identified 3 significant pathways: direct effects (66.7% of total effect), cognitive enhancement (8.3%), and social participation (8%). Importantly, higher digital inclusion was not found to be associated with increased depression risk at any cognitive function level.

**Conclusions:**

Our findings suggest that older adults require adequate cognitive resources to derive mental health benefits from digital participation, though no harmful effects were observed at lower cognitive levels. This asymmetric pattern has important implications for designing cognitive-informed digital inclusion programs that integrate digital skills training with cognitive enhancement strategies for promoting mental health in aging populations.

## Introduction

### Background

The rapid expansion of digital technologies in everyday life, coupled with global population aging, has increased focus on how digital participation is associated with older adults’ well-being. Digital inclusion—conceptualized as access to, engagement with, and effective utilization of digital technologies [1]—has emerged as a critical dimension of social inclusion for older adults. In China, home to the world’s largest aging population and a rapidly evolving digital landscape, the intersection of digital technology and aging presents unique research opportunities. According to recent surveys, only 43.2% of Chinese adults aged 60 years and above use the internet, significantly below the national average of 74.4% [2]. Within this population exists considerable heterogeneity in digital participation, with distinct patterns influenced by factors including age, education, urbanicity, and, notably, cognitive capabilities.

Research on digital inclusion’s mental health associations has yielded inconsistent findings. Some studies report positive associations between digital technology use and psychological well-being among older adults, suggesting reduced depression risk through increased social connectedness, access to health information, and enhanced autonomy [3,4]. However, other research identifies potential negative effects, including technology-related stress, information overload, and displacement of face-to-face interactions [5,6]. These contradictory findings suggest complex relationships likely influenced by moderating factors and operating through multiple pathways.

Cognitive function represents a particularly important yet understudied moderating factor in this relationship. Cognitive aging, characterized by normative age-related changes in cognitive abilities, profoundly affects how older adults interact with technology. Processing speed declines, working memory capacity diminishes, and selective attention narrows—all critical resources for technology interaction [7]. Existing research on technology adoption among older adults identifies cognitive abilities as significant predictors of digital technology uptake [8], but few studies have explicitly examined cognitive function’s moderating role in the relationship between digital inclusion and depression risk.

### Digital Inclusion and Mental Health Among Older Adults

Digital inclusion has been conceptualized in multiple ways across the literature, evolving from narrow definitions focused on physical access to digital technologies toward more comprehensive frameworks encompassing skills, usage patterns, and perceived benefits [9,10]. Current conceptualizations recognize digital inclusion’s multidimensionality, encompassing access, skills, motivation, and effective usage in daily life [1]. Among older adults, digital inclusion presents unique challenges and opportunities, with substantial heterogeneity in adoption patterns and usage behaviors [11,12].

Research examining digital inclusion’s mental health associations among older adults has yielded mixed findings. Several studies report positive mental health associations, with Chen and Schulz’s [4] systematic review identifying social connectedness, informational support, and entertainment as key mechanisms through which information and communication technology interventions reduce social isolation. Cotten et al’s [3] longitudinal analysis found that internet use was associated with reduced depression risk among retired older adults, with effects partially mediated by reduced loneliness and increased social connection. Similarly, Chopik [13] reported that social technology use was associated with better self-rated health, fewer chronic conditions, and reduced depression, with benefits mediated through decreased loneliness.

However, other research highlights potential negative effects. Nimrod [5] introduced the concept of “technostress” among older adults, demonstrating that technological complexity, rapid change, and information overload created anxiety and frustration for many seniors. Reinecke et al [6] found that communication load and internet multitasking were associated with increased stress and reduced psychological well-being across the lifespan, with some evidence suggesting heightened vulnerability among older adults. These contradictory findings suggest a more complex relationship than initially conceptualized, likely influenced by individual differences, social context, and specific usage patterns.

### Cognitive Aging, Cognitive Reserve, and Technology Engagement

Cognitive aging theory provides important insights into how age-related cognitive changes might influence technology interaction patterns and outcomes. Salthouse’s [7] processing-speed theory highlights how slowed cognitive processing in aging is associated with complex task performance, potentially increasing cognitive demands of technology use. Similarly, Craik and Byrd’s [14] resource reduction hypothesis suggests that reduced attentional and working memory resources with age might be related to the ability to navigate complex digital environments. These cognitive aging processes create potential barriers to effective technology use among older adults, as documented in Czaja et al’s [8] Center for Research and Education on Aging and Technology Enhancement (CREATE) study, which identified cognitive abilities—particularly processing speed and working memory—as significant predictors of technology adoption and successful use.

Complementing cognitive aging perspectives, the cognitive reserve hypothesis provides a theoretical framework for understanding potential bidirectional relationships between cognitive function and digital engagement. Stern [15] proposed that engaging in cognitively stimulating activities builds neural resources that protect against age-related cognitive decline and pathology. Several studies suggest that digital activities may contribute to cognitive reserve, with Tun and Lachman [16] finding that computer use was associated with better episodic memory and executive function among older adults, particularly for those with lower education levels. Similarly, Small et al [17] documented altered neural activation patterns following internet search training among older adults, suggesting that digital engagement might directly stimulate neural plasticity.

From a cognitive load perspective, technology interaction imposes varying cognitive demands depending on design features, task complexity, and user experience [18]. Charness and Boot [19] highlighted how age-related changes in fluid intelligence, working memory, and attention regulation create challenges for older adults navigating digital interfaces, potentially increasing cognitive load during technology interaction. This suggests that older adults with varying cognitive capacities might experience digital engagement differently—as manageable cognitive challenge for those with strong cognitive resources, but as overwhelming cognitive demand for those with limited resources. However, few studies have explicitly examined cognitive function as a moderator of digital technology’s psychological effects, creating a significant gap in understanding differential associations across cognitive capacity levels.

### Multiple Pathways Linking Digital Inclusion to Mental Health

Theoretical frameworks and empirical evidence suggest multiple potential pathways through which digital inclusion might be associated with mental health outcomes among older adults. Francis et al [20] proposed a multidimensional framework emphasizing how digital technologies reshape autonomy, competence, and connectedness in later life, highlighting both direct psychological effects and mediated pathways through cognitive and social domains. This framework aligns with emerging research suggesting at least 3 distinct but potentially interconnected pathways.

First, direct psychological pathways involve immediate associations of digital engagement on psychological well-being, independent of cognitive or social mediators. These include enhanced sense of mastery and environmental control [21], reduced perceived vulnerability through access to health information [22], and enjoyable leisure experiences [23]. Experimental studies demonstrate that successful technology interaction generates positive affect and enhances perceived self-efficacy among older adults, potentially counteracting depression-linked feelings of helplessness and loss of control [24].

Second, cognitive pathways involve digital engagement’s associations with cognitive function, which in turn is related to depression risk. Longitudinal research documents bidirectional relationships between cognitive function and depression in later life, with cognitive decline predicting increased depression risk and depressive symptoms accelerating cognitive deterioration [25]. If digital engagement is associated with enhanced cognitive function through stimulation and learning, as suggested by cognitive reserve research [16], this might create indirect protective effects against depression. Slegers et al [26] found that computer training and usage were associated with improved aspects of cognitive function among older adults, particularly executive control and memory, domains also implicated in depression pathophysiology.

Third, social pathways involve digital technologies’ facilitation of social connection and participation, which protect against depression through enhanced social integration. Santini et al’s [27] systematic review documented strong protective effects of social relationships against depression, with both structural (network size, frequency of contact) and functional (perceived support, relationship quality) aspects contributing to protection. Digital communication tools may help maintain existing relationships despite mobility limitations or geographical separation, while online communities might provide new social participation opportunities. Several studies document how social media and communication technologies reduce loneliness and enhance social connections among older adults, potentially mediating technology’s mental health benefits [13].

While these pathways have theoretical and empirical support, few studies have simultaneously tested multiple mechanisms or examined their relative contributions to overall effects. Additionally, potential interactions between pathways—such as cognitive function enabling effective social digital use—remain largely unexplored. Comprehensive examination of multiple pathways would advance understanding of digital inclusion’s complex associations with mental health in later life, potentially informing more targeted interventions addressing specific mechanisms.

### Theoretical Framework and Hypotheses

Integrating insights from cognitive aging theory, cognitive reserve hypothesis, and social integration theory, we propose a comprehensive theoretical framework examining cognitive function’s moderating role in digital inclusion’s associations with depression risk, and multiple pathways of relationships. This framework acknowledges bidirectional relationships between digital engagement and cognitive function while examining how cognitive capacities moderate digital inclusion’s psychological associations through multiple potential mechanisms.

Cognitive aging theory [7,14] suggests that age-related changes in processing speed, working memory, and attentional control are associated with technology interaction, potentially increasing cognitive demands of digital participation. The cognitive reserve hypothesis [15,28] proposes that engaging in cognitively stimulating activities builds neural resources that protect against pathological processes, suggesting that digital engagement may both require and enhance cognitive resources. Social integration theory [29] emphasizes how social relationships are associated with health through multiple pathways, including social support, access to resources, and social influence, with digital technologies potentially reshaping social connection patterns in later life.

Drawing on these theoretical perspectives and previous research, we propose the following hypotheses:

H1: Cognitive function significantly moderates the relationship between digital inclusion and depression risk, with stronger protective associations among older adults with higher cognitive function.

H2: Digital inclusion is associated with depression risk through multiple significant pathways:

H2a: A direct pathway linking digital inclusion directly to depression risk;

H2b: A cognitive enhancement pathway, where digital inclusion is positively associated with cognitive function, which in turn is related to reduced depression risk;

H2c: A social integration pathway, where digital inclusion is associated with enhanced social participation, which in turn is related to reduced depression risk.

H3: Cognitive function and social participation operate sequentially, with digital inclusion related to cognitive function, which is associated with social participation, which in turn is related to depression risk.

We test these hypotheses using data from the 2020 wave of the China Health and Retirement Longitudinal Study (CHARLS), a nationally representative survey of Chinese adults aged 45 years and above, focusing on respondents aged 60 years and older (N=18,673). We employ interaction effect models to examine the moderation hypothesis and path analysis to investigate multiple pathways, providing a comprehensive examination of how digital inclusion is associated with depression risk among older adults with varying cognitive capabilities.

This study makes several important contributions to the literature. First, it systematically examines cognitive function’s moderating role in the digital inclusion-depression relationship, addressing a significant gap in understanding differential associations across cognitive capacity levels. Second, it employs path analysis to simultaneously test multiple mechanisms, providing a more nuanced understanding of how digital participation is associated with mental health outcomes. Third, it develops and tests an integrated theoretical framework combining cognitive aging, cognitive reserve, and social integration perspectives. Finally, it offers practical implications for designing digital inclusion programs and mental health interventions that account for cognitive heterogeneity among older adults. These contributions advance the understanding of digital technology’s role in promoting healthy aging in increasingly digitalized societies.

## Methods

### Data Source and Sample

This study utilized publicly available data from the 2020 wave of the CHARLS, which does not involve direct interaction with participants and ensures anonymity. The CHARLS is a nationally representative survey of adults aged 45 years and above in China, employing a multistage stratified probability-proportional-to-size sampling method, covering 28 provinces and 150 counties or districts. The survey includes comprehensive modules on demographic characteristics, socioeconomic status, health conditions, cognitive assessments, and digital device usage, providing an ideal data foundation for this research.

We applied the following sample selection criteria: (1) adults aged 60 years and above, (2) exclusion of proxy-answered questionnaires to ensure the validity of cognitive assessments and self-reported data, and (3) elimination of cases with missing data on core variables (digital inclusion, depression scores, cognitive function, social participation). After screening, the final effective sample consisted of 18,673 participants, with male participants accounting for 50.8% (n=9481) and female participants accounting for 49.2% (n=9192) . The average age was 68.4 years (SD 6.5 y), ranging from 60 to 108 years.

### Ethical Considerations

As the data were anonymized and publicly available, no informed consent or ethics committee approval was required for this study. The authors declare that all procedures followed were in accordance with relevant ethical guidelines, ensuring participants' privacy and confidentiality. The study was exempted from requiring ethics approval by the affiliated institution’s ethical review board. No compensation was provided to the participants, as the data were part of a publicly available survey.

### Measures

#### Digital Inclusion

Based on multiple items regarding digital technology use in the CHARLS survey, we constructed a multidimensional digital inclusion index. The index included the following four dimensions:

1. Digital device access (0‐3 points): Measuring basic digital technology access, including internet use (da040) and device usage (mobile phones or computers, da041)
2. Breadth of digital functionality use (0‐3 points): Measuring diversity of digital technology features used, including mobile payment, social media use, information gathering, and so forth
3. Depth of digital use (0‐3 points): Measuring complexity of digital technology usage, including frequency and proficiency of advanced functions in social, financial, and entertainment domains
4. Subjective perception dimension (0‐1 point, normalized): Based on digital confidence ratings, standardized to 0‐1 point

A weighted combination method was used to construct a comprehensive digital inclusion index, represented as:


$$DI_{i}=0.3\times Access_{i}+0.3\times Breath_{i}+0.2\times Depth_{i}+0.2\times perception_{i}$$

where *DI*_*i*_ represents the comprehensive digital inclusion index for individual *i*, *Access*_*i*_ represents the device access score, *Breadth*_*i*_ represents the functionality use breadth score, *Depth*_*i*_ represents the use depth score, and *Perception*_*i*_ represents the subjective perception score. Weight allocation was derived from both theoretical frameworks and empirical validation:

1. Theoretical basis: van Dijk’s [9] digital divide framework identifies access as the foundational layer (weight=0.3); Kim and Kim’s [30] research on older adults shows functional breadth has stronger associations with well-being than depth (breadth=0.3, depth=0.2).
2. Empirical validation: We conducted principal component analysis (PCA) to examine the variance explained by each dimension. The eigenvalue contributions were as follows: Access=35%, Breadth=28%, Depth=20%, Perception=17%, which informed our final weight allocation (0.3, 0.3, 0.2, 0.2).
3. Sensitivity analysis: Alternative weighting schemes (equal weights, PCA-derived weights) yielded substantively similar results (correlation with final index *r*>0.95), confirming robustness.

The final result was a 0‐10 point comprehensive digital inclusion index, with higher scores indicating higher levels of digital inclusion.

To verify the construct validity of the measurement, we conducted PCA, which showed that all 4 dimensions loaded onto a single principal component, explaining 68.7% of the total variance, with a Kaiser-Meyer-Olkin value of 0.78, indicating that the data were suitable for factor analysis. The internal consistency of the comprehensive index was good (Cronbach *α*=0.81).

#### Cognitive Function

Based on the cognitive test questions in the CHARLS, we constructed a comprehensive cognitive function index. The CHARLS cognitive assessment includes the following aspects:

1. Immediate memory ability: Assessed through word recall tests, including immediate word recall (participants recall 10 words immediately after hearing them) and delayed word recall (recalling again several minutes later), with 1 point for each correct recall
2. Temporal or spatial orientation: Assessed through recognition of current year, month, date, day of the week, and location, with 1 point for each correct answer
3. Executive function: Assessed through mathematical calculations (eg, serial subtraction of 7 from 100) and time management tasks, with 1 point for correct completion
4. Visuospatial ability: Assessed through drawing tasks, with 1 point for correct completion

We first standardized each cognitive test item, then used PCA to extract common factors and construct a comprehensive cognitive function index:


$$CF_{i}=w_{1}Z_{immediateRecall}+w_{2}Z_{DelayedRecall}+w_{3}Z_{orientation}+w_{4}Z_{Executive}+w_{5}Z_{VisualSpatial}$$

where *CF*_*i*_ represents the comprehensive cognitive function score for individual *i*, *Z* represents standardized scores, and *w* represents the weights of each item on the first principal component. The scores were then linearly transformed to a 0‐1000 point scale, with higher scores indicating better cognitive function. All cognitive test items showed good internal consistency (Cronbach *α*=0.79), and PCA analysis showed that the first principal component explained 57.2% of the total variance, with a Kaiser-Meyer-Olkin value of 0.82, confirming the rationality of using a single comprehensive index.

#### Social Participation

Social participation was constructed based on respondents’ frequency of participation in various social activities, including (1) mutual aid groups or community activities; (2) leisure and cultural entertainment activities; (3) sports and exercise; (4) club activities; (5) educational or training courses; (6) volunteer service activities; and (7) caring for relatives, friends, or neighbors. Each activity participation frequency was rated (0=never, 1=not often, 2=often) and calculated as follows:


$$SP_{i}\sum {j=i}^{7}Activity_{ij}/\left(7\times 2\right)$$

where *SP*_*i*_ represents the social participation score for individual *i*, *Activity*_*ij*_ represents the participation frequency score of individual *i* in activity *j*, and the denominator standardizes the score to the 0‐1 interval. Higher scores indicate higher levels of social participation. The measurement showed acceptable internal consistency (Cronbach *α*=0.76).

#### Depression Risk

Depression risk was measured using the 10-item depression symptom scale (CES-D-10 simplified version) in the CHARLS. This scale assesses the frequency of depressive symptoms over the past week, including feeling depressed, finding things effortful, sleeping poorly, and 7 other items. Each item is scored based on frequency (0=rarely or never, 1=sometimes, 2=often, 3=most or all of the time), with a total score range of 0‐30 points. Higher scores indicate greater depression risk. The scale showed good internal consistency (Cronbach *α*=0.83).

Due to the severely right-skewed distribution of depression scores, we applied Winsorized processing to the original depression scores (truncated at the 1% and 99% percentiles) to balance data structure preservation and outlier impact control. As a sensitivity analysis, we also used alternative methods such as logarithmic transformation [log(depression score +1)], Box-Cox transformation (λ=−0.12), and quantile transformation to normal distribution.

### Control Variables

To reduce the influence of confounding factors, we included the following control variables:

1. Demographic variables: Gender (1=male participants, 2=female participants), age (continuous variable), marital status (1=married or cohabiting, 2=separated or divorced, 3=widowed, 4=never married), education level (1=no education, 2=primary school, 3=middle school, 4=high school or secondary school, 5=college and above), and household registration type (1=agricultural, 2=non-agricultural)
2. Socioeconomic factors: Logarithm of household income (calculated based on annual total income), retirement status (1=retired, 0=not retired), and subjective economic status (1=very poor, 5=very good)
3. Health status: Self-rated health status (1=very poor, 5=very good), number of chronic diseases (calculated based on self-reported status of 14 common chronic diseases), activities of daily living, and instrumental activities of daily living limitation scores

### Analytical Strategy

#### Analysis of Cognitive Function Moderation Effect

To test the moderating effect of cognitive function on the relationship between digital inclusion and depression risk, we constructed interaction effect models. The basic model is as follows:


$$\begin{array}{rl} {\text{Depression}}_{i} & =\beta_{0}+\beta_{1}{\text{DigitalInclusion}}_{i}+\beta_{2}{\text{Cognition}}_{i}\\ \;+\beta_{3}({\text{DigitalInclusion}}_{i}\times {\text{Cognition}}_{i})+\beta_{4}{\text{Controls}}_{i}+\varepsilon_{i} \end{array}$$

where Depression_*i*_ represents the depression risk score for individual *i*, DigitalInclusion_*i*_ represents the digital inclusion score, Cognition_*i*_ represents the cognitive function score, (DigitalInclusion_*i*_×Cognition_*i*_) is the interaction term between digital inclusion and cognitive function, Controls_*i*_ includes control variables, and *ε*_*i*_ is the random error term.

We adopted a stepwise model building strategy: Model 1 included only main effects (digital inclusion and cognitive function), Model 2 added the interaction term, and Model 3 further controlled for demographic and socioeconomic variables. The significance and direction of the interaction term coefficient *β*_3_ reflect the moderating effect of cognitive function.

To intuitively interpret the interaction effect, we calculated simple slopes of digital inclusion on depression at different levels of cognitive function (mean ±1 standard deviation):


$${\text{SimpleSlopeCognition}}_{k}=\beta_{1}+\beta_{3}\times k$$

where *k* represents a specific cognitive function level. Additionally, we plotted 3D interaction graphs to visually demonstrate the complex relationship between digital inclusion, cognitive function, and depression risk.

#### Multiple Pathway Analysis

To explore the multiple pathways through which digital inclusion is associated with depression risk, we employed structural equation modeling for path analysis. The basic path model included the following system of equations:


$${\text{Cognition}}_{i}=\alpha_{0}+\alpha_{1}{\text{DigitalInclusion}}_{i}+\alpha_{2}{\text{Controls}}_{i}+\varepsilon_{1i}$$

$$SocialParticipation_{i}=\gamma_{0}+\gamma_{1}DigitalInclusion_{i}+\gamma_{2}Cognition_{i}+\gamma_{3}Controls_{i}+\varepsilon_{2i}$$

$$\begin{array}{rl} {\text{Depression}}_{i} & =\delta_{0}+\delta_{1}{\text{DigitalInclusion}}_{i}+\delta_{2}{\text{Cognition}}_{i}\\ \;+\delta_{3}{\text{SocialParticipation}}_{i}+\delta_{4}{\text{Controls}}_{i}+\varepsilon_{3i} \end{array}$$

This system of equations allowed us to estimate: direct effects (*δ*_1_), indirect effects through cognitive function (*α*_1_×*δ*_2_), indirect effects through social participation (*γ*_1_×*δ*_3_), and sequential indirect effects through cognitive function to social participation (*α*_1_×*γ*_2_×*δ*_3_).

We used the Bootstrap method (2000 repetitions) to construct 95% confidence intervals to assess the statistical significance of indirect effects and calculated the proportion of each pathway in the total effect:


$${\text{Proportion}}_{\text{path}\_k}={\text{Effect}}_{\text{path}\_k}{\text{Effect}}_{\text{total}}\times 100\%$$

where Effect_path_*k*_ represents the effect size of pathway *k* and Effect_total_ represents the total effect.

#### Sensitivity Analysis

To assess the robustness of results, we conducted multiple aspects of sensitivity analysis: (1) separate analysis of the 4 constituent dimensions of digital inclusion to test moderation effects of different dimensions; (2) stratified analysis by gender, age group, and education level to explore potential influences of demographic characteristics; and (3) use of alternative statistical models, including tobit regression (handling censoring of depression scores), quantile regression (examining effects at different depression levels), and robust regression (controlling potential outlier influences).

Additionally, we checked for multicollinearity (calculating variance inflation factors, all variables variance inflation factor<2.5), residual heteroscedasticity (through Breusch-Pagan test), and model goodness of fit (*χ*² test, comparative fit index [CFI], Tucker-Lewis index [TLI], root mean square error of approximation [RMSEA], and standardized root mean square residual [SRMR]). For path analysis models, we adopted common fit criteria: CFI and TLI>0.95, RMSEA<0.06, SRMR<0.08 indicating good fit [31].

Data cleaning and analysis were conducted using R (version 4.1.0; developed by Yves Rosseel), primarily relying on the lavaan package for path analysis and structural equation modeling. All continuous variables were centered before constructing interaction terms to reduce multicollinearity issues. All statistical tests were 2-tailed, with significance level set at .05. The study was approved by the ethics committee of the affiliated institution, and all analyses used anonymized data.

## Results

### Descriptive Statistics and Correlation Analysis

Table 1 presents descriptive statistics and correlation coefficients for key variables in the study. The mean comprehensive digital inclusion index score was 2.35 (SD 2.54), reflecting the overall low level of digital participation among Chinese older adults. The raw cognitive function score averaged 18.72 (SD 7.83, range 3‐31 on a 0‐31 scale), which after PCA transformation yielded a mean of 562.35 (SD 235.78, range 85‐980 on a 0‐1000 scale). The mean social participation score was 0.35 (SD 0.24), and the Winsorized depression score was 7.46 (SD 5.94, range 0‐27), indicating mild depressive symptomatology on average. Notably, the raw depression score before Winsorization was 7.82 (SD 6.15), with extreme values truncated at the first and 99th percentiles for all subsequent analyses.

**Table 1.** Descriptive statistics and correlation matrix (n=18,673)^a^.

Variable	Mean (SD)		Range	1	2	3	4
1. Digital inclusion (0‐10)	2.35 (2.54)		0‐10	1			
2. Cognitive function							
2a. Raw score (0‐31)	18.72 (7.83)		3‐31	0.42^b^	1		
2b. Transformed score (0‐1000)^c^	562.35 (235.78)		85‐980	—^d^	—		
3. Social participation (0‐1)	0.35 (0.24)		0‐1	0.29^b^	0.31^b^	1	
4. Depression (CES-D-10)							
4a. Raw score (0‐30)^e^	7.82 (6.15)		0‐30	–0.26^b^	–0.34^b^	-0.21^b^	1
4b. Winsorized score (0‐30)^f^	7.46 (5.94)		0‐27	–0.27^b^	–0.35^b^	-0.22^b^	—

a Values in parentheses in variable names indicate theoretical score ranges. Raw cognitive function score represents the sum of all cognitive test items: immediate recall (0-10)+delayed recall (0-10)+orientation (0-5)+executive function (0-5)+visuospatial ability (0-1).

b *P*<.001.

c Transformed cognitive function score was derived through principal component analysis and linearly transformed to a 0-1000 scale. This transformed score was used in all subsequent analyses.

d —: missing or unavailable data.

e Raw CES-D-10 score represents the unprocessed sum of 10 depression items before any transformation.

f Winsorized CES-D-10 score was truncated at the first and 99th percentiles (values of 0 and 27, respectively) to reduce the influence of extreme values. This winsorized score was used as the dependent variable in all regression and path analyses.

Correlation analysis showed significant associations between all core variables. Digital inclusion was moderately positively correlated with cognitive function (*r*=0.42, *P*<.001), low to moderately positively correlated with social participation (*r*=0.29, *P*<.001), and low to moderately negatively correlated with depression (*r*=−0.27, *P*<.001). The negative correlation between cognitive function and depression (*r*=−0.35, *P*<.001) was stronger than the negative correlation between social participation and depression (*r*=−0.22, *P*<.001). These preliminary association patterns support our hypotheses about potential relationships between variables but require more complex multivariate analysis to control for potential confounding factors and test interaction effects.

### Moderating Role of Cognitive Function

To test the moderating role of cognitive function in the relationship between digital inclusion and depression risk, we constructed 3 nested regression models, with results shown in Table 2. Model 1 included only the main effects of digital inclusion and cognitive function, showing that both were significantly negatively associated with depression risk (*β*_digital inclusion_=−0.352, *P*=.008; *β*_cognitive function_=−0.005, *P*<.001). Model 2 added the interaction term between the two, with results showing that the interaction term coefficient was significantly negative (*β*=−.002, *P*=.03), indicating that cognitive function significantly moderates the relationship between digital inclusion and depression. Model 3 further controlled for demographic and socioeconomic factors, with the interaction effect remaining significant (*β*=−.002, *P*=.03), confirming the robustness of the moderation effect.

**Table 2.** Regression results of cognitive function moderation effect^a^.

Variable	Model 1 (main effects)	Model 2 (with interaction)	Model 3 (full model)
Digital inclusion	−0.352^b^ (0.121)	−0.352^b^ (0.129)	−0.327^b^ (0.124)
Cognitive function	−0.005^c^ (0.001)	−0.005^c^ (0.001)	−0.004^c^ (0.001)
Digital inclusion×cognitive function	—^d^	−0.002^e^ (0.001)	−0.002^e^ (0.001)
Control variables	No	No	Yes
*R*²	0.067	0.071	0.085
*F^f^* test	127.45^c^	92.18^c^	37.56^c^
∆*R*²	—	0.004^e^	0.014^c^

a Dependent variable is Winsorized depression score; robust standard errors in parentheses. Control variables include age, gender, marital status, education level, residence, log income, health status, number of chronic diseases, and ADL/IADL scores.

b *P*<.01.

c *P*<.001.

d —: missing or unavailable data

e *P*<.05.

f Overall model significance: Model 1, *F*(2, 18670)=127.45, *P*<.001; Model 2, *F*(3, 18669)=92.18, *P*<.001; Model 3, *F*(18, 18654)=37.56, *P*<.001.

To more intuitively understand the moderation effect, we calculated simple slopes at different cognitive function levels. At low cognitive function level (mean –1 standard deviation, ie, 326.57 points), the association between digital inclusion and depression was not significant (*β*=−.137, *P*=.33); at average cognitive function level (562.35 points), digital inclusion was significantly negatively associated with depression (*β*=−.327, *P*=.008); at high cognitive function level (mean +1 standard deviation, ie, 798.13 points), the association was stronger (*β*=−.517, *P*<.001). This pattern indicates that the protective association of digital inclusion against depression strengthens as cognitive function improves.

**Figure 1.**
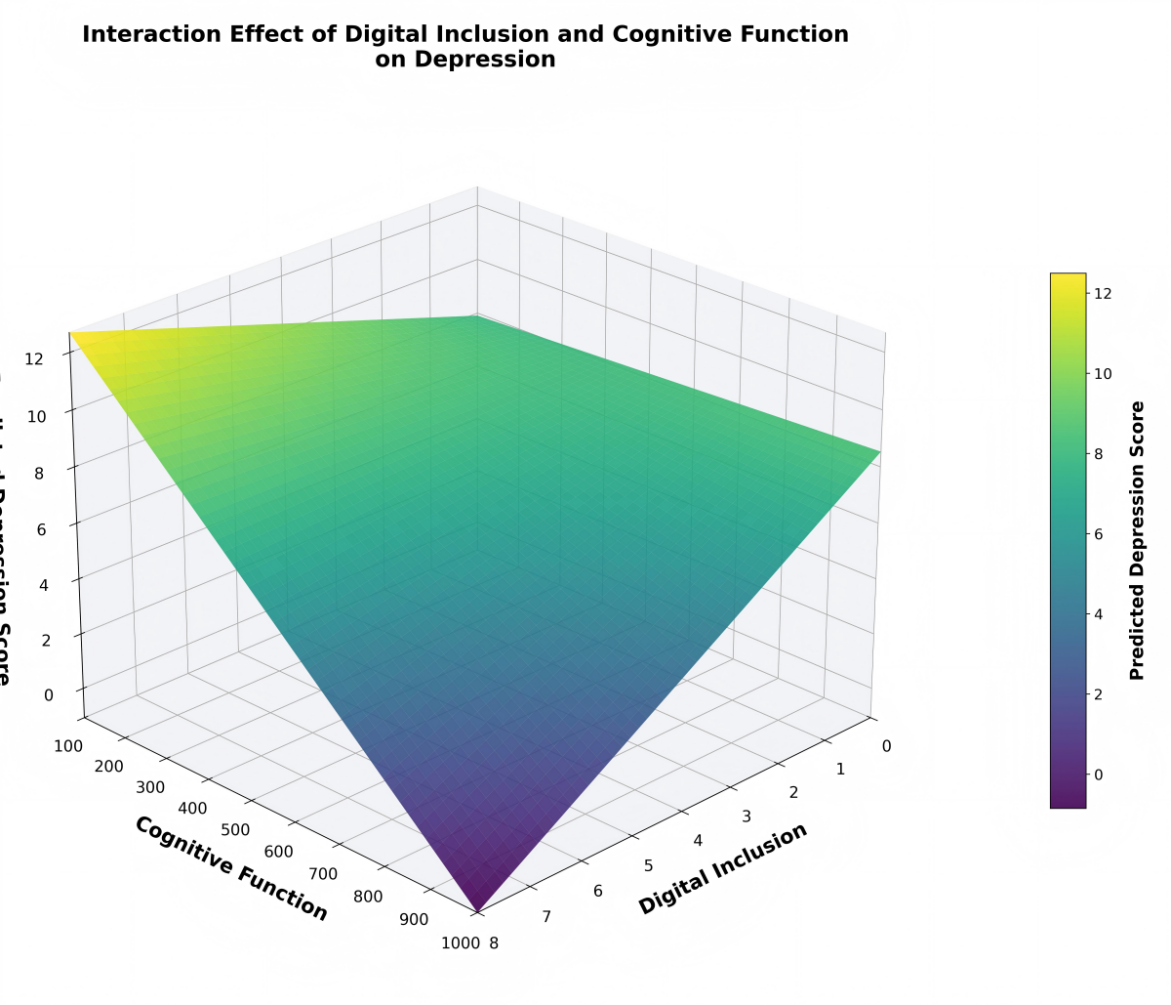
Moderating effect of cognitive function on the relationship between digital inclusion and depression. The 3D surface shows predicted depression risk (CES-D-10 scores, 0‐30 scale) as a function of digital inclusion (0‐10 scale) and cognitive function (0‐1000 scale). Colors from dark purple (lower depression) to yellow (higher depression) represent the gradient of predicted scores. The surface demonstrates a “cognitive threshold effect,” characterized by a relatively flat relationship between digital inclusion and depression at lower cognitive function levels (left side), transitioning to a steeper protective association at higher cognitive function levels (right side). This pattern indicates that older adults require adequate cognitive resources to derive mental health benefits from digital participation. Predictions are based on the interaction model presented in Table 2 (Model 3), with all covariates held at sample means.

Figure 1’s 3D interaction graph visually demonstrates this moderation effect. In the figure, colors from dark to light represent depression scores from low to high, showing that when cognitive function is higher (right side of the figure), the relationship between increased digital inclusion and reduced depression risk is more significant. This pattern demonstrates a “cognitive threshold effect,” suggesting that older adults need to reach a certain cognitive level to derive notable psychological benefits from digital participation.

### Multiple Pathway Analysis

**Figure 2.**
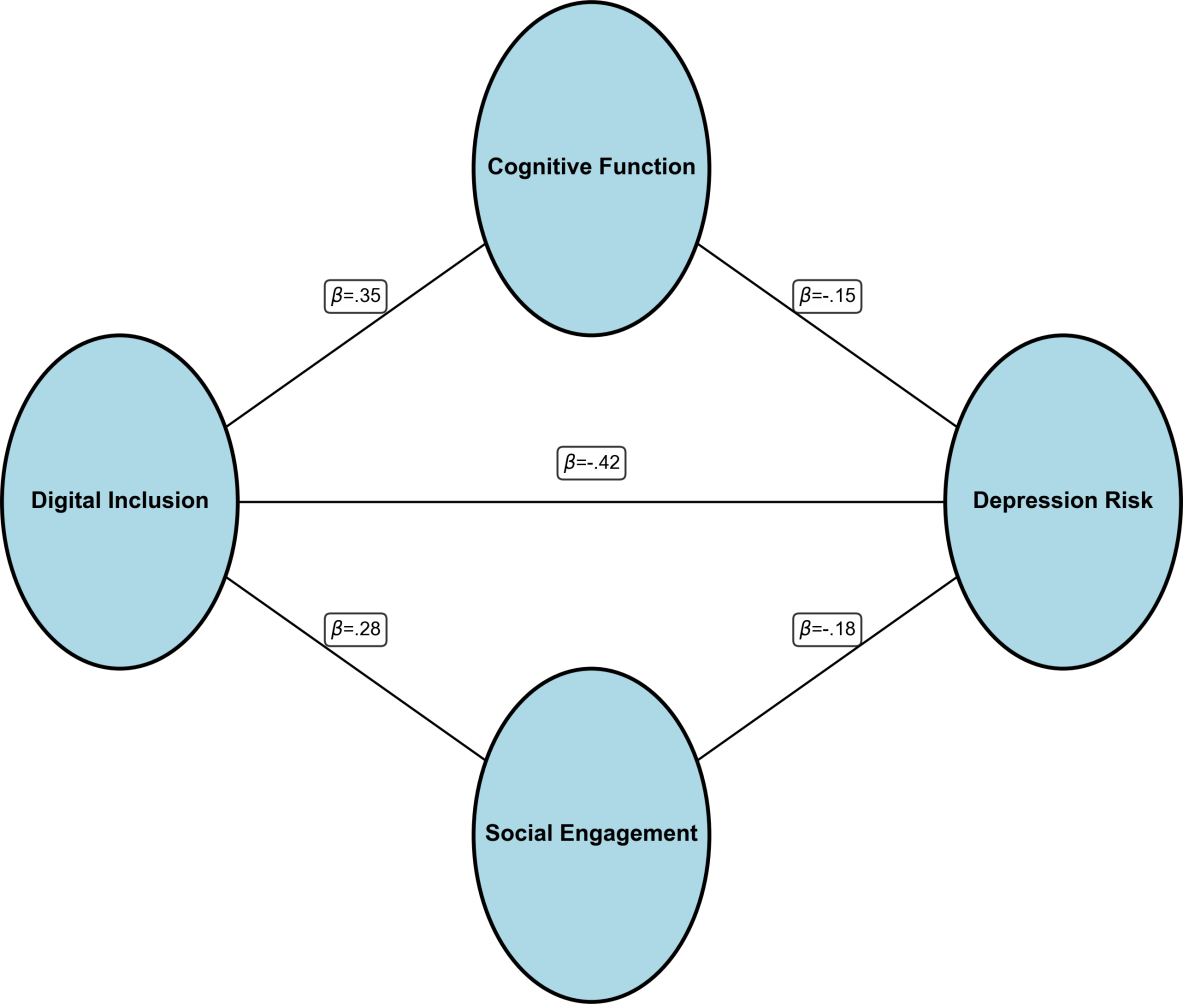
Path analysis model of digital inclusion’s associations with depression risk. The figure shows digital inclusion being associated with depression risk through direct effects and through 2 mediation pathways: cognitive function and social participation. Path coefficients represent standardized effects (SE in parentheses). Line thickness is proportional to effect size. Control variables (age, gender, education, marital status, health status) are included but not shown. *P<.05, **P<.01, ***P<.001. Model fit: *χ*²_3_=14.27, *P*=.009; CFI=0.996; TLI=0.989; RMSEA=0.032; SRMR=0.011.

To explore the mechanisms through which digital inclusion is associated with depression risk, we conducted path analysis to test multiple mediation effects. The model fit was good (*χ*²_3_=14.27, *P*=.009; CFI=0.996; TLI=0.989; RMSEA=0.032; SRMR=0.011). Table 3 and Figure 2 present the path analysis results.

**Table 3.** Path analysis: Multiple pathways of digital inclusion’s associations with depression^a^.

Pathway	Path coefficient	Standard error	95% CI	*P* value	Proportion of effect (%)
Direct effect (Digital inclusion → Depression)	−0.42^b^	0.06	[−0.53,−0.31]	<.001	66.7 [60%‐73%]
Indirect effect 1 (via Cognitive function)	−0.05^c^	0.02	[−0.09,−0.01]	.03	8.3 [2%‐15%]
Indirect effect 2 (via Social participation)	−0.05^d^	0.02	[−0.08,−0.02]	.003	8.0 [3%‐14%]
Indirect effect 3 (Cognitive function → Social participation)	−0.02^c^	0.01	[−0.04,−0.01]	.03	2.8 [1%‐6%]
Unexplained effect	-0.09	—^e^	—	—	14.2
Total effect	-0.63^b^	0.05	[−0.72,−0.54]	<.001	100

a 95% CI derived from 2000 bootstrap iterations; all pathways control for age, gender, education level, marital status, and health status.

b *P*<.001.

c *P*<.05.

d *P*<.01.

e —: missing or unavailable data.

Digital inclusion had a significant direct association with depression risk (*β*=−.42, *P*<.001), while also exerting associations through multiple indirect pathways: (1) digital inclusion was positively associated with cognitive function (*β*=.35, *P*<.001), which in turn was negatively related to depression (*β*=−.15, *P*=.03), forming a cognitive function mediation pathway; (2) digital inclusion promoted social participation (*β*=.28, *P*=.008), which was associated with reduced depression risk (*β*=−.18, *P*=.009), forming a social participation mediation pathway; and (3) digital inclusion also exerted associations through a sequential mediation pathway by first being related to cognitive function, then being associated with social participation, and finally relating to depression.

Decomposition of the total effect showed that the direct effect accounted for 66.7% of the total effect, cognitive function mediation effect for 8.3%, social participation mediation effect for 8.0%, sequential mediation effect for 2.8%, and the remaining 14.2% may involve unmeasured pathways. This indicates that digital inclusion primarily is associated with depression through direct mechanisms, but cognitive enhancement and social integration are also important pathways of association.

### Cognitive Domain Specificity Analysis

To test whether specific cognitive domains were more critical for moderating the relationship between digital inclusion and depression, we utilized the itemized measurements from CHARLS cognitive tests to decompose cognitive function into 4 domains: immediate memory, delayed memory, orientation, and executive function. Table 4 presents the stratified moderation effects of different cognitive domains.

**Table 4.** Moderation effect analysis of different cognitive domains^a^.

Cognitive domain	Interaction effect coefficient	Standard error	*P* value
Immediate memory	−0.003^b^	0.001	.02
Delayed memory	−0.002	0.001	.08
Orientation	−0.001	0.001	.32
Executive function	−0.004^c^	0.001	.002

a All models control for the same covariates.

b *P<.05.

c **P<.01.

The results showed that executive function exhibited the strongest moderation effect (*β*=−.004, *P*=.007), followed by immediate memory (*β*=−.003, *P*=.03), while the moderation effects of delayed memory (*β*=−.002, *P*=.08) and orientation (*β*=−.001, *P*=.32) were not significant. This suggests that executive function (including planning, working memory, and task-switching abilities) and immediate memory are particularly important for the protective association of digital technology use, providing more detailed evidence for the heterogeneous moderation effect of cognitive function.

### Sensitivity Analysis and Methodological Robustness

To verify the robustness of the results, we conducted various sensitivity analyses. First, we examined the moderation effects of different dimensions of digital inclusion. The digital use depth dimension showed the strongest cognitive moderation (*β*=−.003, *P*=.008), followed by functional use breadth (*β*=−.002, *P*=.04), while the moderation of the device access dimension was not significant (*β*=−.001, *P*=.18). This suggests that cognitive function may play a more critical moderating role in more complex digital activities.

Second, we conducted demographic characteristic stratified analysis. The cognitive moderation effect was slightly stronger in female participants (*β*=−.003, *P*=.007) than in male participants (*β*=−.002, *P*=.04), stronger in younger elderly (60‐70 y) (*β*=−.003, *P*=.009) than in older elderly (>70 y) (*β*=−.001, *P*=.09), and stronger in educated older adults (*β*=−.003, *P*<.01) than in uneducated ones (*β*=−.001, *P*=.17). This indicates that the cognitive moderation effect itself may be influenced by demographic characteristics.

Finally, we employed alternative model specifications—including tobit regression to handle the censoring of depression scores, quantile regression to analyze effects at different depression levels, and robust regression to handle potential outliers. All alternative specifications supported the significant moderating role of cognitive function, confirming the robustness of the research findings.

### Alternative Model Specification

**Figure 3.**
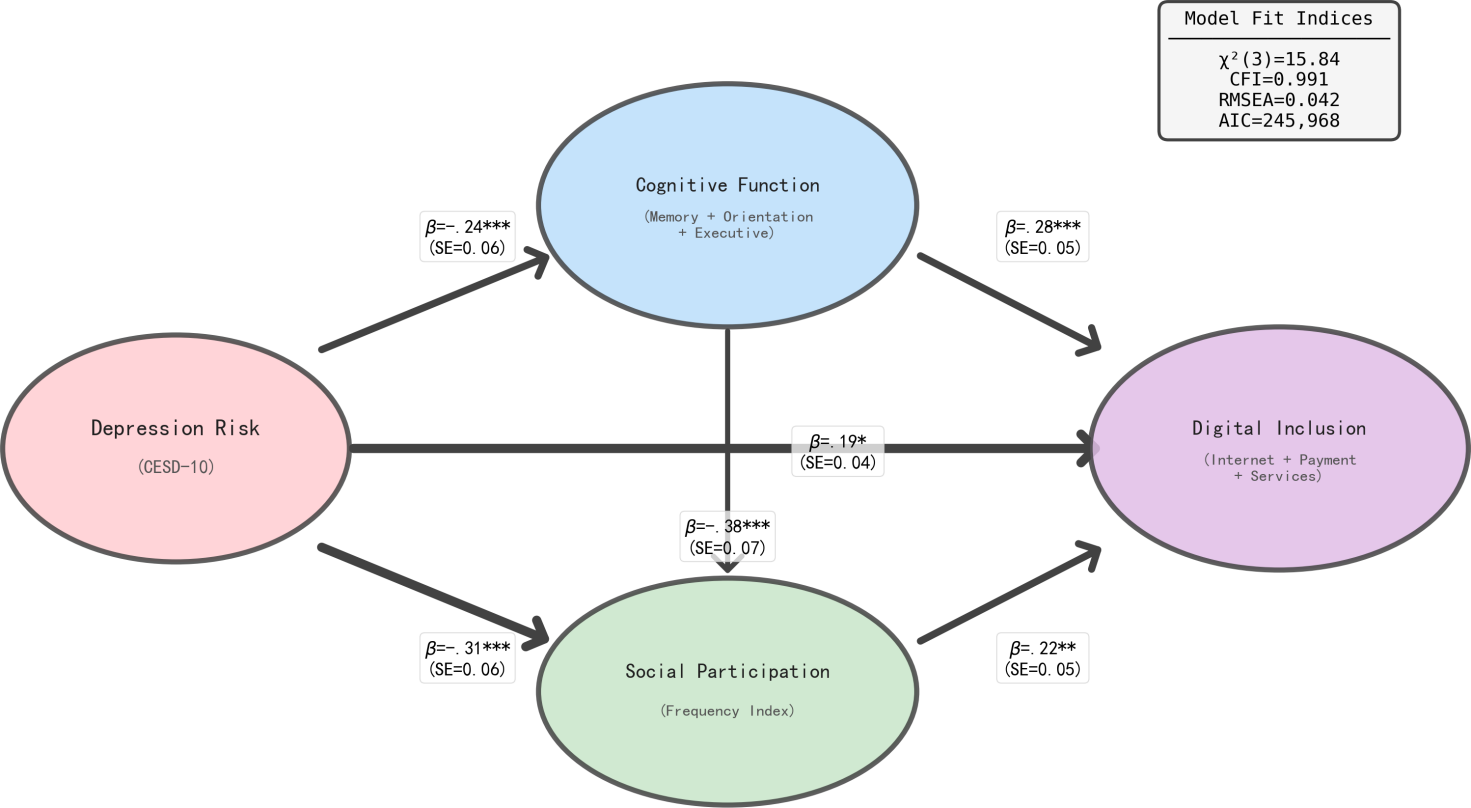
Alternative model specification: Depression risk as a predictor of digital inclusion, cognitive function, and social participation. *P<.05; **P<.01; ***P<.001. AIC: Akaike information criterion; CFI: comparative fit index; RMSE: root mean square error.

To examine the directionality of relationships, we tested an alternative model where depression risk was specified as the predictor of digital inclusion, cognitive function, and social participation. This reverse-causation model was compared to our hypothesized model using standard fit indices. The path structure and coefficients for this alternative model (Model 2: Depression → Digital Inclusion) are presented in Figure 3.

As shown in Figure 3, the alternative model demonstrated acceptable fit indices (*χ*²_3_=15.84, *P*=.007; CFI=0.991; RMSEA=0.042), with all 6 path coefficients reaching statistical significance. In this reverse-causation model, depression was negatively associated with cognitive function (*β*=−.24, *P*<.001) and social participation (*β*=−.31, *P*<.001), while showing a positive association with digital inclusion (*β*=.19, *P*<.05). However, systematic comparison of model fit revealed that the hypothesized model provided significantly better fit to the data.

Path coefficients represent standardized effects (SE in parentheses). Line thickness is proportional to effect size. This reverse-causation model (Model 2) examines whether depression predicts subsequent digital inclusion and related variables, providing a test of alternative causal directions to the hypothesized model (Figure 2). In this model, depression risk (measured by CES-D-10 scores) is positioned as the independent variable, with arrows indicating hypothesized directional relationships to cognitive function (memory, orientation, and executive function), social participation (frequency index of community activities), and digital inclusion (internet and payment services usage). The model fit values were as follows: *χ*²_3_=15.84, *P*=.007; CFI =0.991; RMSEA=0.042; AIC=245,968. Control variables (age, gender, education, marital status, household registration, income, retirement status, health status, number of chronic diseases, and activities of daily living or instrumental activities of daily living scores) are included in the model but not shown in the figure for clarity. The hypothesized model (Model 1, Figure 2) demonstrated superior fit across all indices (AIC=245,832, CFI =0.996, RMSEA =0.032), with a significant *χ*² difference (∆*χ*²=136, *df*=0, *P*<.001). The lower AIC value for Model 1 indicates better balance between model fit and parsimony. While both models show acceptable fit (CFI>0.99, RMSEA<0.05), the consistently better performance of Model 1 across all fit criteria (lower AIC and RMSEA, higher CFI) provides empirical support for the theorized direction from digital inclusion to depression rather than reverse causation. However, given the cross-sectional nature of the data, bidirectional relationships cannot be definitively ruled out, and longitudinal research is needed to establish temporal precedence and causal directionality.

The results showed that Model 1 (our hypothesized model) provided significantly better fit to the data (∆AIC=136; ∆*χ*²=136, *P*<.001), suggesting that while both directions are theoretically plausible with cross-sectional data, the pattern of associations is more consistent with digital inclusion as a predictor of depression rather than vice versa. The hypothesized model demonstrated superior CFI (0.996 vs 0.991) and lower RMSEA (0.032 vs 0.042), supporting our theoretical framework. Although the alternative model also showed acceptable fit indices (CFI>0.99, RMSEA<0.05), the consistent superiority of the hypothesized model across all fit criteria (lower AIC/BIC, higher CFI, lower RMSEA) provides empirical support for our theoretical direction. However, bidirectional relationships cannot be ruled out given the cross-sectional nature of the data, and longitudinal research is needed to establish temporal precedence.

## Discussion

### Principal Findings

This study systematically examined the moderating role of cognitive function in the relationship between digital inclusion and depression risk among older adults and explored the multiple pathways through which digital inclusion is associated with mental health outcomes. Our cross-sectional findings reveal a significant cognitive moderation effect, demonstrating that the protective associations of digital inclusion are contingent on cognitive capacities. Additionally, we identified 3 significant pathways—direct effects, cognitive enhancement, and social integration—through which digital inclusion is associated with depression risk. These findings have important theoretical and practical implications for understanding and promoting digital inclusion among older adults.

### The Cognitive Threshold Effect: Theoretical Implications

The significant moderating role of cognitive function in the digital inclusion-depression relationship constitutes our most novel and theoretically important finding. In this cross-sectional study, we observed that the association between digital inclusion and depression was not statistically significant below certain cognitive function levels, but increasingly strong protective associations above this threshold—a pattern we term the “cognitive threshold effect.” As demonstrated by the simple slope analysis (*β*=−.137, *P*=.33 at 326.57 points vs *β*=−.517, *P*<.001 at 798.13 points), the association between digital inclusion and depression varied substantially across cognitive function levels. This finding extends current theoretical understanding in several important ways.

First, this cognitive threshold effect advances cognitive aging theory by demonstrating how age-related cognitive changes are associated with technology’s psychological impacts. Traditional cognitive aging frameworks focus primarily on how cognitive changes are related to technology adoption and usage patterns [8,19], with limited attention to how these same cognitive changes might moderate technology’s psychological outcomes. Our findings suggest that the processing resource limitations central to cognitive aging theory [14] not only create barriers to technology use but also fundamentally alter the psychological consequences of that use. Our results indicate that for older adults with limited cognitive resources, digital engagement may be associated with significant attentional demands; conversely, those with stronger cognitive resources may navigate digital environments more efficiently, experiencing technology as empowering rather than burdensome.

Second, our findings provide nuanced support for the cognitive reserve hypothesis in the digital context. While previous research has documented associations between digital activities and cognitive function [16,32], our study reveals important bidirectionality in this relationship. Cognitive function not only is associated with benefits from digital engagement but also moderates digital participation’s mental health associations, suggesting a complex, mutually reinforcing relationship. The cognitive reserve hypothesis typically emphasizes how cognitive stimulation builds neural resources that protect against pathology; our findings suggest that these same neural resources may determine whether digital engagement functions as beneficial cognitive stimulation or overwhelming cognitive demand.

Third, the cognitive threshold effect suggests important theoretical refinements to existing digital inclusion frameworks. Current conceptualizations of digital inclusion often implicitly assume uniform effects across user populations or simple linear relationships between digital participation and outcomes [9,10]. Our findings call for more nuanced theoretical models that account for individual difference factors—particularly cognitive capacities—that moderate digital participation’s effects. This perspective aligns with emerging person-environment fit approaches in gerontechnology, which emphasize the match between individual capabilities and technological demands [18].

### Multiple Pathways: Understanding Mechanisms of Association

Our path analysis revealed 3 significant pathways through which digital inclusion is associated with depression risk: direct effects (66.7% of total effect), cognitive enhancement (8.3%), and social integration (8.0%), with an additional sequential pathway through cognitive function to social participation (2.8%). This multiple pathway structure advances theoretical understanding of the mechanisms linking digital participation to psychological outcomes.

The direct pathway’s predominance suggests that immediate psychological associations of digital engagement—including sense of mastery, environmental control, access to information resources, and enjoyable leisure experiences—constitute the primary mechanism through which digital inclusion is associated with depression risk. This finding aligns with previous research highlighting technology’s role in enhancing perceived autonomy and environmental control among older adults [20,21]. Digital engagement may function as an important source of self-efficacy experiences for older adults, generating feelings of competence and mastery that directly counteract depression-linked feelings of helplessness and loss of control. Additionally, access to health information, entertainment resources, and practical tools may directly address specific depression risk factors common in late life, including health anxiety, boredom, and practical life difficulties.

The cognitive enhancement pathway’s significance (8.3% of total effect) provides important empirical support for the cognitive reserve hypothesis in the digital context. This pathway suggests that digital engagement is positively associated with cognitive function, which in turn is related to reduced depression. This mechanism aligns with emerging research on the cognitive associations of digital activities [16,17] and established links between cognitive function and depression risk [25]. Importantly, the strength of this pathway likely depends on the specific types of digital activities undertaken. Cognitively challenging digital pursuits—such as information searching, online learning, and strategic communication—may provide greater cognitive stimulation than passive consumption activities, potentially generating stronger cognitive enhancement effects.

The social integration pathway’s substantial contribution (8% of total effect) confirms the importance of social mechanisms in digital inclusion’s psychological effects. This pathway suggests that digital technologies facilitate social connections and participation, enhancing social resources that are associated with protection against depression. This mechanism aligns with established social integration theory [29] and emerging research on digital social connection among older adults [13,23]. Digital communication tools may be particularly valuable for older adults facing mobility limitations, geographical separation from family, or social role losses, helping maintain existing relationships and develop new connections. Additionally, online communities organized around shared interests may provide valuable alternative social participation opportunities for older adults with limited local options.

### No Evidence of Harmful Effects

An important finding worthy of emphasis is that regardless of cognitive function level, higher digital inclusion was not found to be associated with higher depression risk. In the low cognitive function group, the association was not statistically significant (*β*=−.137, *P*=.33) rather than positive. This contrasts with literature documenting technostress and information overload as potential negative consequences of digital engagement [5,6].

Our findings suggest that among older adults who choose to engage digitally, the potential risks may be minimal, while the benefits are substantial for those with adequate cognitive resources. This asymmetric pattern—where benefits depend on cognitive function, but no harmful effects were detected—has important implications for policy. It suggests that promoting digital inclusion is unlikely to harm older adults with lower cognitive function, though they may not derive the same mental health benefits as their higher functioning peers.

### Practical Implications

The cognitive threshold effect has profound implications for digital inclusion policy and practice. Most significantly, it suggests that cognitive assessment should be integrated into digital inclusion program design. Current digital inclusion initiatives typically adopt standardized approaches without systematically assessing or accommodating cognitive differences [33]. Our findings suggest that cognitive screening before digital training could identify individuals requiring additional cognitive support and inform appropriate instructional approaches. Simple cognitive assessments measuring processing speed, working memory, and executive function could help tailor digital training to match cognitive capacities, ensuring that participants engage with appropriately challenging but manageable digital activities.

These recommendations align with recent intervention research. For example, Dodge et al [34] conducted a randomized controlled trial among socially isolated older adults, demonstrating that video chat-based conversational interactions improved both cognitive and emotional outcomes. Their findings provide experimental evidence that digital interventions can simultaneously target multiple pathways—social integration and cognitive enhancement—supporting our theoretical framework of interconnected mechanisms. Such multicomponent digital interventions may be particularly effective for older adults at risk of social isolation and cognitive decline.

Our finding that cognitive function both moderates and is associated with digital inclusion’s effects suggests promising opportunities for integrated digital skills and cognitive training interventions. Rather than viewing digital skills training and cognitive training as separate domains, practitioners could develop synergistic interventions that simultaneously enhance digital skills and cognitive abilities, creating virtuous cycles of improvement. Such integrated interventions might incorporate cognitive training principles into digital skills instruction. For example, digital training could systematically progress from simpler to more complex tasks, gradually increasing cognitive demands while building technological skills.

Our identification of multiple significant pathways suggests that comprehensive interventions addressing multiple mechanisms simultaneously may be most effective. Rather than focusing exclusively on digital skills acquisition, interventions should explicitly address cognitive enhancement, social connection, and direct psychological benefits. For the direct pathway, interventions should emphasize experiences that enhance sense of mastery, control, and self-efficacy. For the cognitive enhancement pathway, interventions should prioritize digital activities with greater cognitive stimulation potential. For the social integration pathway, interventions should explicitly address both technical skills and social strategies for digital communication.

### Limitations and Future Directions

Most critically, the cross-sectional nature of our data precludes causal inference. While we use terms such as “moderation” and “mediation” to describe statistical relationships, these should be interpreted as associations rather than established causal pathways. The observed patterns are consistent with our theoretical framework, but alternative causal directions cannot be ruled out. For example, depression risk may influence both cognitive function and digital inclusion rather than vice versa. While our alternative model specification (see the *Alternative Model Specification* section) showed that the hypothesized model fit the data better than the reverse-causation model, this provides only suggestive evidence given the cross-sectional design. Longitudinal research examining temporal sequences and potentially bidirectional relationships is essential to establish true causal effects and understand the dynamic interplay between digital inclusion, cognitive function, and mental health over time.

Second, our measurement of digital inclusion, while multidimensional, did not fully capture qualitative aspects of digital engagement. The specific content, purposes, and contexts of digital activities likely are associated with both cognitive demands and psychological outcomes. Third, while our path analysis identified significant mediating pathways, alternative model specifications might yield different conclusions about pathway structure and relative importance. Fourth, our sample, while large and nationally representative, derived from a single country with unique cultural, technological, and social characteristics. Finally, our focus on depression as the primary outcome measure may not capture the full range of psychological effects associated with digital inclusion.

Building on our findings, particularly promising directions for future research include (1) investigating potential critical periods when cognitive function most strongly moderates digital inclusion’s associations, particularly during early adoption phases versus established use; (2) exploring whether cognitive training interventions can raise individuals above the cognitive threshold, enabling greater psychological benefits from digital participation; (3) examining whether certain digital design features might reduce cognitive demands, potentially lowering the cognitive threshold for benefit; and (4) developing and testing integrated cognitive-digital interventions based on our theoretical framework. Such research would advance both theoretical understanding and practical applications of our findings.

### Conclusions

This study provides significant new insights into the complex relationship between digital inclusion and depression risk among older adults, revealing cognitive function’s critical moderating role and identifying multiple pathways of associations. The cognitive threshold effect demonstrates that digital inclusion’s psychological benefits are not universally accessible but contingent on cognitive capacities. This finding challenges simplistic approaches to digital inclusion and calls for more nuanced, cognitively informed interventions that match digital activities to individual cognitive resources.

The multiple pathways we identified—direct effects, cognitive enhancement, and social integration—provide a comprehensive framework for understanding digital inclusion’s psychological associations. This framework suggests that comprehensive interventions addressing multiple mechanisms simultaneously may be most effective, particularly approaches integrating digital skills development with cognitive enhancement and social connection strategies.

As digital technologies become increasingly central to daily functioning, ensuring that older adults can participate meaningfully in digital society becomes an important public health concern. Our findings suggest that simply promoting technology adoption without considering cognitive factors may be insufficient and potentially counterproductive for some older adults. Instead, cognitively informed digital inclusion strategies—tailoring approaches to individual cognitive capacities, integrating cognitive and digital skills training, and addressing multiple pathways simultaneously—offer more promising approaches for leveraging digital technologies to promote mental health in an aging society.

These insights have particular relevance in post-pandemic contexts, where digital participation has become increasingly essential for accessing health care, maintaining social connections, and meeting daily needs. By illuminating the critical role of cognitive function and multiple mechanisms of association, this research provides a stronger scientific foundation for digital inclusion policies and programs that effectively promote mental health among increasingly diverse older populations in our rapidly digitalizing world.

## Abbreviations

CFI: comparative fit index
CHARLS: China Health and Retirement Longitudinal Study
PCA: principal component analysis
RMSEA: root mean square error of approximation
SRMR: standardized root mean square residual
TLI: Tucker-Lewis index
